# Current concepts in the assessment and management of multiligament injuries of the knee

**DOI:** 10.1051/sicotj/2021058

**Published:** 2021-12-06

**Authors:** Waldo Scheepers, Vikas Khanduja, Michael Held

**Affiliations:** 1 Department of Orthopedic Surgery, Groote Schuur Hospital, Orthopedic Research Unit, University of Cape Town 7925 Cape Town South Africa; 2 Consultant Orthopedic Surgeon, Addenbrooke’s – Cambridge University Hospital Cambridge CB2 0QQ United Kingdom

**Keywords:** Multiligament knee injuries, Knee dislocations, Assessment, Management, Review

## Abstract

Multiligament knee injuries (MLKIs), though rare, pose significant challenges to the patient and surgeon. They often occur in the setting of high-velocity trauma and are frequently associated with concomitant intra- and extra-articular injuries, the most immediately devastating of which is vascular compromise. A detailed evaluation is required when acute or chronic MLKIs are suspected, and stress radiography, MRI and angiography are valuable adjuncts to a thorough clinical examination. Surgical treatment is widely regarded as superior to non-surgical management and has been demonstrated to improve functional outcome scores, return to work, and return to sport rates, though the incidence of post-traumatic osteoarthritis remains high in affected knees. However, acceptable results have been obtained with conservative management in populations where surgical intervention is not feasible. Early arthroscopic single-stage reconstruction is currently the mainstay of treatment for these injuries, but some recent comparative studies have found no significant differences in outcomes. Recent trends in the literature on MLKIs seem to favour early surgery over delayed surgery, though both methods have distinct advantages and disadvantages. Due to the heterogeneity of the injury and the diversity of patient factors, treatment needs to be individualised, and a single best approach with regards to the timing of surgery, repair versus reconstruction, surgical technique and surgical principles cannot be advocated. There is much controversy in the literature surrounding these topics. Early post-operative rehabilitation remains one of the most important positive prognostic factors in surgical management and requires a dedicated team-based approach. Though outcomes of MLKIs are generally favourable, complications are abundant and precautionary measures should be implemented where possible. Low resource settings are faced with unique challenges, necessitating adaptability and pragmatism in tailoring a management strategy capable of achieving comparable outcomes.

## Introduction

A knee dislocation (KD) is defined as tibiofemoral disarticulation, but spontaneous reduction and KDs with intact cruciate ligaments have added complexity to this definition [1]. The term multiligament knee injury (MLKI) is therefore used with less ambiguity when two or more of the four main knee ligaments are injured: these are the anterior cruciate ligament (ACL), posterior cruciate ligament (PCL), posterolateral corner (PLC) and posteromedial corner (PMC) [2]. KDs are rare, with an estimated 0.02–0.2% of orthopaedic injuries, but the true incidence is likely to be underestimated due to spontaneous reduction or missed injuries in the polytrauma patient [3, 4].

In 1824, Sir Astley Cooper made the observation about knee dislocations: “Of this, I have only seen one instance, and I conclude it, therefore, to be a rare occurrence; and there are scarcely any accidents to which the body is more liable which more imperiously demand immediate amputation than these.” [5] Consequently, KDs were historically associated with detrimental sequelae such as loss of life and limb, and initial management focused on conservative strategies [6–9]. This has changed as surgical techniques and intricate anatomic and biomechanical knowledge evolved [10]. Yet, some of these historical findings are still applicable in certain settings.

The scarcity and heterogeneity of this injury make adequately powered prospective clinical trials in a similar setting challenging, resulting in a paucity of high-level evidence [11]. Consequently, there is much controversy in the literature about surgical versus non-surgical management, repair versus reconstruction, surgical techniques and the ideal timing of MLKI surgery. This creates the need for interpretation and contemporary insight into the specific considerations that need to be taken into account when tailoring an individualised, evidence-based approach to managing a multiple-ligament injured knee. Therefore, the aim of this article is to review, debate, and critically interrogate the current literature to provide an overview of the background, assessment, management, and outcomes of MLKIs.

## Etiology

KDs can result from high-, low- and ultra-low-velocity injuries. High-velocity injuries are usually caused by motor-vehicle accidents, falls from a significant height or severe crush injuries and are more likely to have associated injuries [12, 13]. Most low-velocity knee dislocations occur during sporting activities or falls from less than approximately 1.5 m and typically have better overall outcomes [12, 13]. Ultra-low velocity KDs mostly occur in obese patients and are often sustained during activities of daily living [12]. With the current obesity pandemic, these injuries have become more frequent, leading to a demographic peak of the obese elderly population, besides young patients with high-energy injuries [14]. The obese patients are especially challenging to examine [11], which can be detrimental as they present a higher rate of associated injuries and post-operative complications than non-obese patients [15].

## Classification

The Schenck classification system is most commonly used to categorise knee dislocations [11]. It describes the anatomical pattern of ligamentous disruption and has been modified to include specifiers for neurovascular injuries. More detail can be added for each of these grades (i.e. via Müeller charts), which could potentially aid surgical planning [11] and add prognostic value for clinical outcome [16]. The positional Kennedy classification and other energy-based classification systems have been found inadequate for communication or guiding management as they are limited in describing the severity and pattern of ligamentous injury – especially in cases of spontaneous reduction [17].

## Associated injuries

There is a high incidence of intra-and extra-articular injuries in MLKIs. The presence of meniscal or chondral injuries has been reported in up to 76% of cases (55% and 48%, respectively) [18]. Associated vascular injuries are common and potentially devastating, with amputation rates of 12% [3, 19], which rises to 80% if limb ischaemia exceeds 8 h [20]. In a systematic review of 23 studies and 907 patients, an 18% incidence of vascular injuries in KDs was found, with a rate of 32% in Schenck KDIIIL patterns [19]. The popliteal artery is most frequently affected in 83.6% of cases, followed by the tibial artery in 7.54% of cases [20]. The incidence of associated neurological injury varies greatly in literature (5–59%), with a 2014 systematic review by Medina et al. including 862 patients estimating it at 25% [19]. A retrospective review of the American College of Surgeons National Trauma Data Bank, including 6454 patients, reported an incidence of 6.2% [20]. The common peroneal nerve (CPN) is most often affected (53.3%) and is associated with posterolateral corner injuries in 21.6% of cases [21]. The resultant loss of antigravity strength is potentially disabling [22]. Furthermore, the presence of an open injury significantly complicates management as they often occur in the setting of high-velocity polytrauma with substantial damage to surrounding soft-tissues [23]. Open injuries occur in 13.6% of KDs [20] and carry a greater infection risk of up to 43% [23]. They also constitute a risk factor for vascular injury [21] and raise the amputation rate to 15.6% [20].

## Evaluation

**Figure 1:**
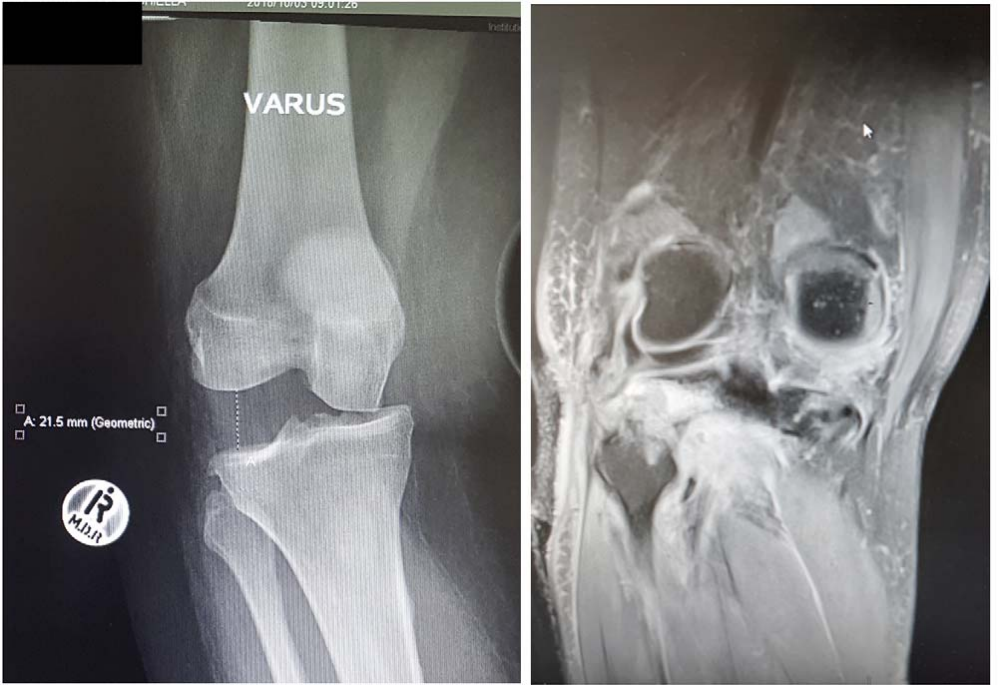
Imaging in a patient with a suspected MLKI. Left: AP varus stress radiograph shows a large lateral joint gap indicative of a complete disruption of the LCL, associated disruption of cruciate ligaments should be suspected. Right: The injury can also be seen on coronal T2 MRI with disruption of the posterolateral complex.

A detailed history and clinical examination should precede available imaging techniques. Magnetic resonance imaging (MRI) has become the gold standard in the evaluation of injured structures [24], but stress radiography remains useful in both acute and chronic injuries, especially if MRI is not available (Figure 1) [25].

### Acute injuries

The initial assessment must abide by advanced trauma life support (ATLS) principles as MLKIs are frequently complicated by comorbid polytrauma [26]. A thorough neurovascular exam is always warranted, and an Ankle Brachial Index (ABI) should be performed [27] and serially monitored as the development of occlusive thromboses formed by intimal flap tears may only be revealed with time [28]. An ABI < 0.9 or an expanding haematoma indicates angiography [29], and CT or MRI angiography should be considered a first-line modality for diagnosing arterial injury [27]. In many Level 1 centres in high-resource settings, polytrauma patients routinely undergo whole-body CT scans on arrival with CT angiography for suspected KDs. Traditionally, arteriography has been the gold standard for detecting vascular injuries but is expensive, requires arterial puncture and has a complication rate of up to 9% [30]. If an arterial injury is found, acute revascularisation is required, and the limb should be immobilised, ideally in a transarticular external fixator for 2–6 weeks to preserve the integrity of the vascular graft and joint reduction keeping in mind the risk of pin tract infection and joint stiffness [25, 27].

### Chronic injuries

As a result of spontaneous reduction of KDs in up to 50% of cases and concomitant polytrauma, MLKIs are often missed in the acute setting [4]. Here as well, MRI plays a pivotal role in assessing damaged intra-articular structures and should be routinely used when available [25]. Comparative stress radiographs can objectively and dynamically assess the laxity of healed but elongated ligaments which are often overlooked, especially in chronic PCL or MCL tears on MRI scans [27].

Moatshe et al. have devised a grading system of instability of the PCL, LCL, and MCL based on stress radiographs by comparing the injured and uninjured knees (Table 1) [25]. The mechanical axis should also be determined radiographically to detect malalignment prior to ligament reconstruction [25]. If varus malalignment is present in chronic PLC injuries, a corrective osteotomy should be considered prior to reconstruction to prevent excessive graft tension and failure [25].

**Table 1:** Evaluation of posterior, varus and valgus knee instability using stress radiographs [25].

Poster instability			Varus instability		Valgus instability	
Kneeling stress radiographs (PTT)	Injury	Grade of PCL injury	Varus stress test	Injury	Valgus stress test	Injury
≤ 7 mm	Normal or partial tear	I	≤ 2.6 mm	Normal or partial tear	≤ 3.1 mm	Normal or partial tear
8–11 mm	Complete PCL tear	II	2.7–3.9 mm	Isolated LCL tear	3.2–9.7 mm	Complete sMCL tear
≥ 12 mm	Combined ligament injury	III	≥ 4 mm	Complete PLC injury	≥ 9.8 mm	Complete tear of all medial structures

LCL, lateral collateral ligament; PCL, posterior collateral ligament; PLC, posterolateral corner; PTT, posterior tibial translation; sMCL, superficial medial collateral ligament.

## Surgical versus non-surgical management

Surgical treatment of MLKIs is widely regarded as superior to non-surgical management. Dedmond and Almekinders performed a meta-analysis that assessed surgically and non-surgically treated knees and demonstrated a better range of motion, flexion contractures and Lysholm scores in the surgically treated group [10]. An evidence-based review by Peskun and Whelan found that surgical versus non-surgical management improved Lysholm scores, IKDC scores, and Tegner activity scores. The differences in IKDC and Tegner activity scores were not statistically significant. No significant differences were found in ROM between groups, but improvements in instability as assessed by KT-1000 TM measurements were found in the operative cohort. Return to employment and return to a comparable level of athletic competition were considerably higher with surgery [31]. A systematic review by Levy et al. found improved IKDC scores, return to work rates and return to full sport rates in the surgical cohorts [2]. Plancher et al. retrospectively evaluated 50 knees, of which 31 were treated surgically and 19 conservatively and found that the surgical cohort was significantly less likely to develop severe radiographic degenerative changes (47.4% and 88%, respectively) [32, 33]. Most smaller retrospective studies have found similar improvements in surgically treated patients when assessing range of motion and functional outcome scores (Table 2) [34–38].

**Table 2:** Outcome comparison of surgical versus nonsurgical treatment [2, 10, 31, 34–38].

Study	Design	Number of patients		Lysholm Score		IKDC Score (% Good/excellent)		Tegner activity score		Range of motion (°)		Loss of flexion (°)		Return to work (%)		Return to sport (%)	
		S	NS	S	NS	S	NS	S	NS	S	NS	S	NS	S	NS	S	NS
Dedmond and Almekinders [10]	Meta-analysis	132	74	85.2	66.5					123	108	0.54	3.5	58	50	31	14
Levy et al. [2]	Systematic Review	227	107			58	20			126	123	4	3	72	52	29	10
Peskun and Whelan [31]	Systematic Review	855	61	84.3	67.2	61.3	25.0	4.8	2.7					80.9	50	57.8	22.2
Almekinders and Logan [35]	Retrospective Study	6	10							129	108						
Richter et al. [34]	Retrospective cohort	59	18	78	65	24	6	4	3					85	53	56	17
Wong et al. [38]	Retrospective cohort	15	11			75.84	63.71			129	137	6	2			0	0
Ríos et al. [37]	Retrospective cohort	21	5	77	40	76	0										
Demirağ et al. [36]	Retrospective cohort	6	6	84.6	74					116	72						

Abbreviations: S, Surgical; NS, Non-surgical.

Although surgical treatment has been demonstrated to be superior to non-surgical treatment, a pragmatic approach should be taken, and surgery might not always be feasible in low-resource settings (LRS). Closed reduction, immobilisation with an external fixator or cast for 4–6 weeks and a period of non-weight-bearing has been reported to attain acceptable outcomes in cases where surgery is not feasible, but regular radiographic evaluation to ensure that reduction is maintained is essential [39–41]. Range of motion can subsequently be improved with manipulation under anaesthesia or arthroscopic adhesiolysis after immobilisation [40]. However, data supporting conservative management is old, and advances in surgical methods have affirmed the superiority of operative treatment. Non-operative treatment should only be considered when surgical intervention is unavailable and in special populations such as the morbidly obese, patients with vascular or open injuries, patients unable to attend rehabilitation, the elderly and comorbid-burdened patients [25, 40].

## Repair versus reconstruction

Multiligament injured knees can either be repaired with sutures or reconstructed with the use of various grafts. Repair is ideally performed in the acute period (within 3 weeks of the injury), whereas reconstruction can be done early, later or in stages [42]. Staged surgery consists of acute primary repair of collateral ligaments with cruciate reconstruction once improved range of motion has been obtained, and good outcomes have been reported for range of motion and stability [43, 44]. Systematic reviews by Jiang et al. and Mook et al. found improved subjective outcomes and range of motion with staged procedures [45, 46]. However, a meta-analysis by Frosch et al. compared suture repair versus reconstruction of cruciate ligaments and found that a two-staged procedure wherein collateral ligaments are repaired without addressing the cruciate ligaments cannot be recommended [47]. A staged reconstruction can cause altered joint kinematics and increase the risk of graft failure, thus single-stage reconstruction is advocated by some authors to avoid this complication while facilitating early mobilisation and mitigating joint stiffness [25]. Though acceptable results have been reported with both repair and reconstruction, repair of the MCL generally does not offer benefit over nonoperative treatment [47]. In many studies that include bicruciate injuries, the repair cohort underwent PCL suturing, and the ACL was left untreated, making the accurate comparison of repair versus reconstruction challenging [47]. The heterogeneity of knee injuries and lack of high-level evidence on the matter necessitates individualised consideration when choosing an approach [48].

Mariani et al. demonstrated that reconstruction yielded better stability, range of motion, functional outcome scores and return to pre-injury activities [49]. However, this data is more than 2 decades old. A meta-analysis by Frosch et al. reported good or excellent IKDC or Lysholm scores with both methods and found no significant difference between the two [47]. A systematic review by Levy et al. showed similar functional outcome scores with repair and reconstruction, but stability, ROM and return to pre-injury activity levels was higher in the reconstruction cohort [2]. A combined repair-reconstruction approach has been advocated if collateral ligaments and extra-articular structures are affected [50]. Historically, posterolateral corner injuries treated with reconstruction have lower reoperation rates than when repaired [2, 25, 42]. Stannard et al. found failure rates of 37% with PLC repair compared to 9% failure with reconstruction. It should be noted that about half of the patients in both the repair and reconstruction groups (48.5% and 54.5%, respectively) had a hinged external fixator post-operatively [51]. Similarly, Levy et al. compared LCL/PLC repair and reconstruction and found failure rates of 40% and 6%, respectively [52]. Therefore reconstruction is widely accepted as gold-standard, although some recent prospective and retrospective studies have found no significant differences in outcomes between methods [48]. There also seems to be no clear difference comparing repair to reconstruction regarding medial collateral ligaments and posteromedial corner injuries [42]. Repair is generally favoured for avulsion fractures [40, 42, 48] and has been advocated when ligaments are torn at their insertions [53]. A recent resurgence of interest in repair has been brought about with the introduction of internal bracing, in which primary repairs are synthetically augmented [40, 54, 55]. LRS may benefit from this method as surgical time is reduced by avoiding graft harvesting, and the stability of a primary repair is enhanced [40]. Equivalent outcome results have been reported with this method [54, 56]. A recent descriptive cross-sectional scenario-based survey compared approaches to MLKI management between surgeons in emerging markets and developing nations (EMDNs) and developed economic nations (DENs). It found that surgeons from EMDNs preferred conservative management and delayed staged reconstruction with autograft and often did not have access to a physiotherapist. DENs surgeons favour early, single-stage arthroscopic ligament reconstruction [57].

## Timing

Conflicting data exist about the ideal timing of surgery for MLKIs. Additionally, there is ambiguity about the time frame defining “early” and “delayed” surgery [42, 48, 58]. Early surgery has typically been described as an intervention within 3 weeks of the injury, when soft-tissue integrity is intact, and tissue planes are still definable [48], whereas late surgery refers to intervention after 4–6 weeks [58]. Advantages of early surgery include earlier restoration of normal joint kinematics and earlier mobilisation, which may improve functional outcomes, though there is an increase in arthrofibrosis and knee stiffness [40, 42]. Delayed reconstruction allows extra-articular structures to heal, improving ROM and potentially avoiding additional unnecessary surgery, but is associated with a higher risk of further chondral or meniscal damage [40, 42]. Early post-operative rehabilitation is the most important positive prognostic factor, regardless of the timing of surgery [40]. Thus delayed reconstruction is an attractive option in LRS as structures may heal independently and negate unnecessary interventions, and where proper rehabilitation is not readily available. Though controversial, overall recent trends in literature seem to favour early surgery with improved or similar outcomes compared to delayed surgery [2, 42, 44, 48, 58–60].

## Surgical techniques

**Figure 2:**
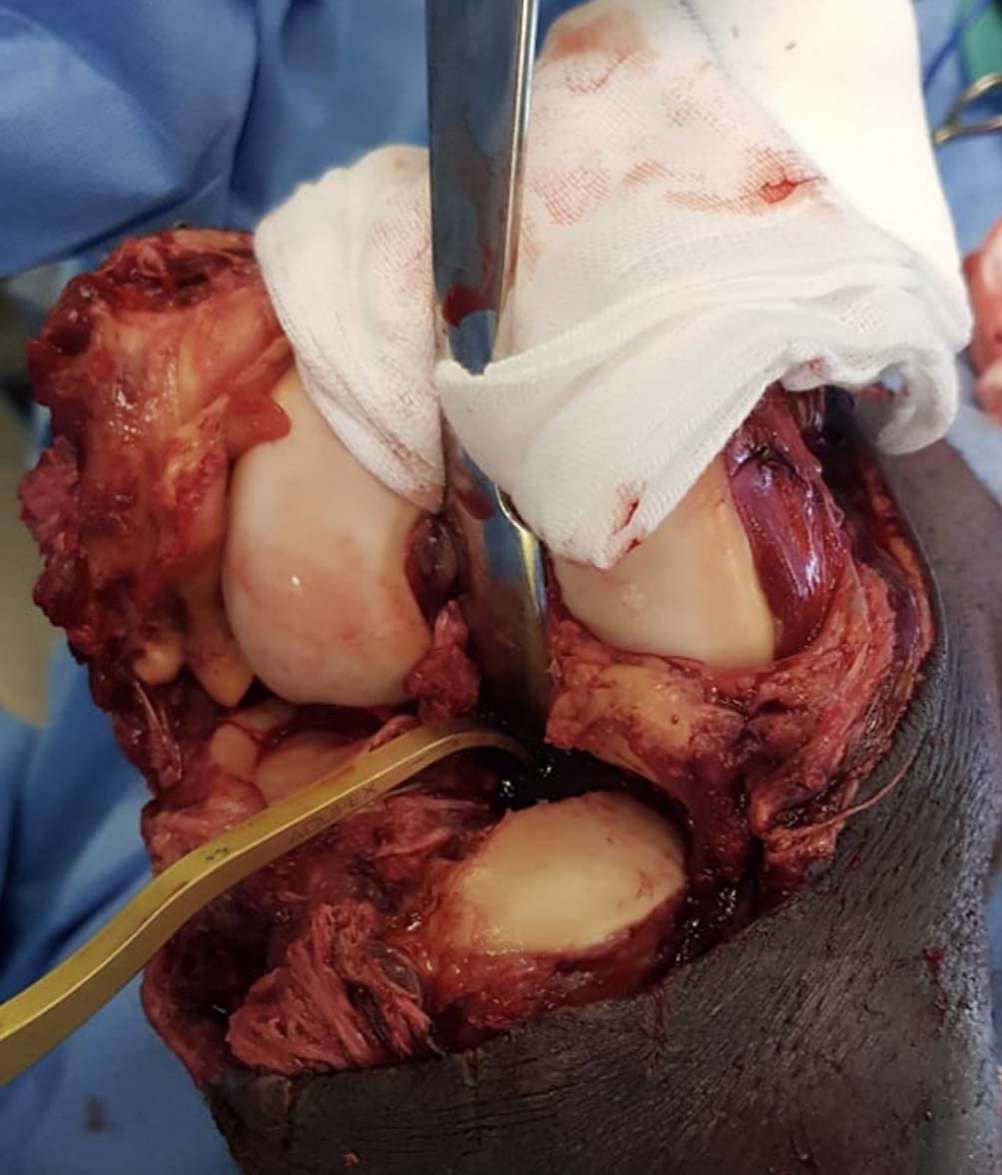
Open surgery in a patient with a traumatic knee arthrotomy and MLKI. Open cruciate surgery done in a patient with an open knee dislocation, patella tendon rupture and large traumatic arthrotomy. The PCL tunnel is drilled under direct vision with a PCL tunnel aimer.

Various surgical techniques have been described and need to be tailored to the patient and thorough pre-operative assessment. Historically, open repair of all ligaments was advocated, an approach that is now outdated [61]. Acute arthroscopic single-stage reconstruction is the current gold-standard treatment for ligamentous injuries but is not always available [40, 62]. Furthermore, if arthroscopy is performed too soon in MLKI, it may result in fluid extravasation and compartment syndrome due to capsular disruption [63]. Therefore, some recommend delaying surgery by 10–14 days to allow swelling to subside and capsular healing [63]. Open surgery is performed where arthroscopy is not feasible (Figure 2).

If reconstruction is chosen, graft selection includes autografts, allografts and synthetic grafts – each with a set of distinct advantages and disadvantages (Table 3) [42, 64]. Fixation is dependent on graft choice, and graft size is another important consideration (Table 4) [64, 65]. Graft selection is dependent on the preference of the surgeon and patient, availability, and the number of injured ligaments to be reconstructed [65].

**Table 3:** A comparison of autografts versus allografts [64].

	Autograft	Allograft
Cost	No additional cost	Additional cost
Availability	Readily available	May not be readily available
Risk	No risk of disease transmission	Risk of disease transmission
	Additional surgical risks	No additional surgical risks
Quality	Good quality	Quality may be reduced if irradiated (relevant in low-resource settings with a high burden of transmissible disease)
Morbidity	Donor site morbidity	No donor site morbidity
Operating time	Less operating time	More operating time
Tissue reaction	Minimal	Variable

**Table 4:** Advantages and disadvantages of different grafts [64, 65].

Graft	Advantages	Disadvantages
Bone – Patella Tendon – Bone	Large grafts	Risk of patella fracture
	Bone-to-bone healing	Anterior knee pain
	Less graft stretching	Quadriceps weakening
	Lower incidence of tunnel widening and rupture	
Quadriceps tendon	Large cross-sectional area	Technically challenging to harvest
	Low donor-site morbidity	
	Bone-to-bone healing on one end	
Peroneus longus tendon	Long graft	Risk of ankle pain and instability
	Large cross-sectional area	
	Simple harvest technique	
Achilles tendon or tibialis anterior allografts	No donor site morbidity	Low quality when irradiated
	Shorter operation time	Risk of disease transmission when not irradiated
	No size limitation	Local bone resorption
		Delayed incorporation
		Additional costs
Hamstring (gracilis and semitendinosus)	No anterior knee pain	Prolonged ligamentization and soft-tissue healing
		Haematoma formation
	Low donor site morbidity	Poor predictability of graft size
	Fast graft acquisition	Weakening of ACL agonists
	Easy graft preparation and passage	Reduced speed in athletes
	High load to failure	

Both the ACL and PCL can be reconstructed with single- or double-bundles techniques. For the ACL, single-bundle reconstruction is currently favoured [40]. Double-bundle reconstruction of the PCL has been shown to better replicate normal knee kinematics and reduce residual posterior translation, although clinical outcomes remain similar [42]. Good results have been reported with single-staged and two-staged procedures, and the ultimate decision is dependent on resources as well as the surgeon’s preference and ability [58]. The distinct advantage of single-stage surgery lies in facilitating early mobilisation and preventing joint stiffness [25]. In vascular repairs, open injuries, gross obesity, or in selected cases with severe instability, an external fixation device can be indicated to achieve initial stability [24].

## Rehabilitation

Post-operative treatment needs to be individualised, and the outcome is dependent on the cooperation of the patient, surgeon and multi-disciplinary team involved. Most experts recommend an initial period of non-weight-bearing for 4–6 weeks, followed by active mobilization and progressive weight-bearing [48]. Early surgery combined with early motion (defined as achieving greater than 30 degrees motion within 3 weeks of surgery) has been found to reduce posterior instability, varus and valgus laxity, flexion loss of more than 10°, extension loss of more than 5° and results in improved outcome scores when compared to early surgery with delayed rehabilitation [66]. However, strength often remains poor at 2 years after surgery for MLKIs with notable deficits in both quadriceps and hamstrings [66]. The importance of rehabilitation and early mobilisation has historically been a cardinal factor in achieving desirable outcomes and remains relevant today [61].

## Outcomes

Although most studies of MLKIs treated surgically consist of small cohorts with short follow-up periods, surgical management of these injuries results in good functional outcomes as assessed by validated scoring systems [10]. However, the outcome can vary with IKDC scores as low as 67 [3] and as high as 82 [67] on medium-term follow-up. The incidence of radiographic osteoarthritis (OA) varies in the literature, with reports of 23%, by Fanelli et al. [68] 42% by Moatshe et al. [33] and 87% by Engebretsen et al. [69] over follow-up periods of 2–12 years. Only 6.8% of the patients in the study by Fanelli et al. eventually underwent total knee arthroplasty [68], with Moatshe et al. demonstrating a similar rate of 7.7% [33]. Regarding revision MLKI reconstruction, Woodmass et al. assessed the outcomes in 23 patients at a mean of 7.5 years follow-up and found average Lysholm and IKDC scores of 79.4 and 74.5, respectively [70]. The Multiligament Quality of Life (ML-QOL) scoring system designed by Chahal et al. has recently gained attention to evaluate MLKIs as it is a disease-specific questionnaire consisting of four relevant subsections – physical, emotional, activity and social subscales [71, 72]. Increased risk of OA is associated with high-energy trauma, age over 30 years and associated cartilage injuries [40]. A systematic review and meta-analysis by Poulsen et al. analysed approximately one million patients with various knee injuries and found a four times greater risk of developing OA with ACL injuries when compared to non-injured knees and a six fold increase with combined ACL and meniscal injuries [73]. In a retrospective cohort study, Richter et al. found that the degree of radiological OA as measured by the Jäger and Wirth Score correlated with the incidence of MCL and LCL ruptures and with knee stability at follow-up, not with the incidence of meniscal damage [34, 40]. However, not all patients with radiological OA have symptoms [74]. Though controversial, several authors have advocated that early surgery reduces the risk of severe OA [74]. In cases of intractable pain and functional limitations caused by severe OA, total knee arthroplasty may offer relief. Poor functional outcomes are also associated with high-energy trauma and patient age over 30 years, and additionally with the repair of medial sided injuries and combined medial and lateral meniscal tears [25].

Everhart et al. performed a systematic review of 21 studies, including 524 patients, to determine overall rates of return to work or sport after MLKI. They found a return to any level of sport was 53.6%, with a return to high-level sport significantly lower at 22–33% [75]. Return to any work was possible for 88.4% of patients, although only 62.1% could do so with minimal modifications [75]. Return to work was lower in patients with Schenck Grade IV and V injuries, as well as in patients with vascular injuries. Obese patients had worse Tegner activity scores when compared to the non-obese (mean scores of 1.7 vs. 4.5) [75].

## Complications

Complications of MLKI are extensive, they can be acute or chronic and may be injury or intervention related. Vascular injuries are easily missed at presentation or caused iatrogenically, with the popliteal artery being placed at risk during PCL reconstruction [76]. Nerve injuries are also common and often result from the injury itself, though the peroneal nerve is at risk of injury during PLC repair and reconstruction [76]. Patients are predisposed to venous thromboembolic events and pulmonary emboli after MLKI surgery, but the risk can be mitigated with routine thromboprophylaxis [76]. Arthrofibrosis after surgery poses another challenge that can be seen in up to 29% of patients after surgery, especially if performed in the acute phase [76]. Twenty one percent of these require manipulation under anaesthesia and potentially arthroscopic or open surgical adhesiolysis [76, 77].

Preventative measures constitute meticulous handling of soft-tissue, arthroscopy, minimising ipsilateral autografts, reduction of post-operative inflammation, and swelling and appropriate rehabilitation [76]. If significant capsular disruption or fascial damage is present, early arthroscopy within 1–2 weeks of the injury may result in fluid extravasation and compartment syndrome [63, 76]. Heterotropic ossification, avascular necrosis and fractures secondary to loss of bone stock with surgical tunnelling and hardware implantation can occur [76]. Recurrent instability is another potential complication [40]. Fanelli et al. evaluated knee stability in a cohort of 44 patients and reported KT1000™ arthrometer side-to-side differences of >5 mm in 16% of cases (7 patients), a similar rate of instability to other studies [68]. Post-operative infection rates are as high as 17% but can be reduced with the routine use of perioperative antibiotics [40]. Diabetics, the obese and patients undergoing prolonged surgery are predisposed to developing an infection [40]. Other intraoperative precautions include avoiding new incisions which cross scars or wounds, preserving skin bridges of at least 10 cm between incisions, careful soft-tissue handling, achieving proper haemostasis before wound closure, minimising tension with wound closure and using drains when needed to prevent haematoma formation [76]. Open injuries carry a much higher rate of post-operative infection, and immediate debridement, irrigation, intravenous antibiotics and external fixation is often warranted to reduce this risk [76].

## Authors’ commentary

**Figure 3:**
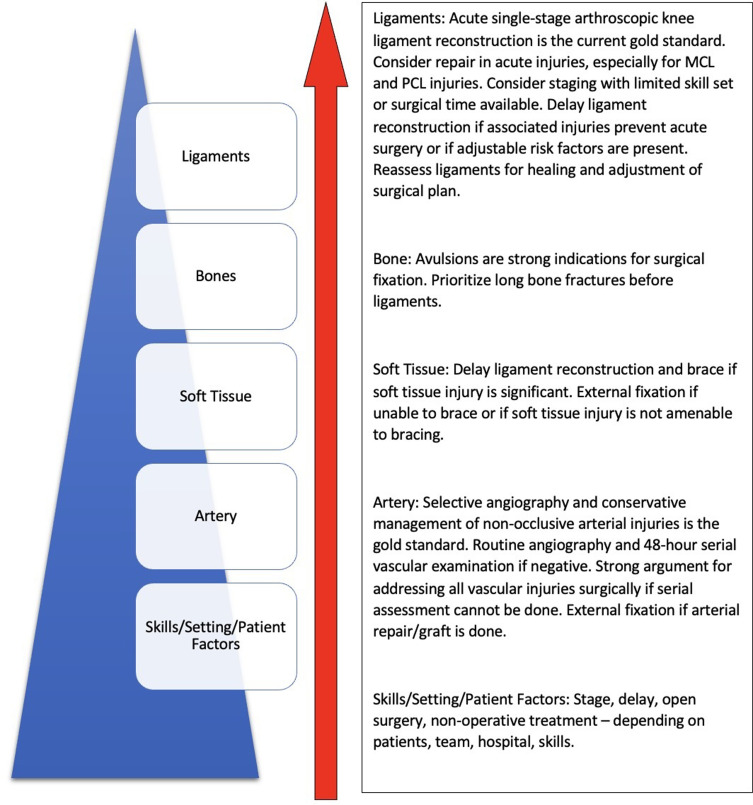
Pyramid of priorities when choosing an approach for knee ligament reconstruction. A summary of arguments for various approaches outlines the considerations when choosing an approach specific to knee ligament reconstruction. The pyramid of priorities in order of importance includes life-threatening injuries, vascular compromise, soft-tissue damage, fractures, and ligamentous injury. Available skills, the setting and patient factors will influence decision making at each step.

As aforementioned, the heterogeneity of MLKIs and variation in patient and environmental factors necessitate an individualised approach when choosing a management plan. Above is a summary of arguments for various approaches that outline the considerations when choosing an approach specific to knee ligament reconstruction. The pyramid of priorities in order of importance includes life-threatening injuries, vascular compromise, soft-tissue damage, fractures and ligamentous injury. Available skills, the setting and patient factors will influence decision making at each step (Figure 3).

## Conclusion

Multiligament knee injuries are challenging entities and are often missed in the presence of polytrauma. A high index of suspicion is required to detect associated injuries with potentially devastating complications. Vascular injuries are common, and a thorough clinical examination and monitoring of the limb are crucial. Angiography is indicated if the ABI is <0.9 or in the presence of an expanding haematoma. Stress radiography plays a pivotal role in evaluating both acute and chronic injuries, pre-and postoperatively. MRI should be used routinely where available. Knee dislocations are fraught with complications, both injury-related and iatrogenic, which can be mitigated with a thorough pre-operative assessment and complication-specific precautions. An open discussion with the patient about their expectations and likely outcomes is essential. Surgical treatment is superior to non-surgical treatment and results in good functional outcome scores, though in special populations and resource-restricted environments, conservative management is an acceptable treatment option. Despite favourable outcomes, the incidence of OA in the multiligament injured knee remains high. The importance of proper post-operative rehabilitation is emphasised repeatedly as a strong positive prognostic factor, and patient motivation remains a cornerstone to success. The heavy burden of MLKIs in LRS requires further consideration to address the unique challenges faced in their context. As evidenced by the literature, intense debate surrounds many aspects of MLKI surgery, such as timing, repair versus reconstruction and optimal tensioning sequences. This stresses the need for future research to produce high-level evidence on the topic, and the rapid evolution of technology and techniques will demand continuous and astute critical assessment.

## Conflicts of interest

The authors declare they have no relevant financial or non-financial conflicts of interest to declare.

## Funding

This research did not receive any specific funding.

## Ethical Approval

Ethical approval was not required.

## Informed Consent

This article does not contain any studies involving human subjects.

## Author Contributions

W. Scheepers – writing original draft, editing; V. Khanduja – writing, reviewing, editing; M. Held – writing, reviewing, editing.
